# Changes of anthropometric indicators of lithuanian first-graders in 2008–2019 according to International Obesity Task Force (IOTF) and World Health Organization (WHO) definitions

**DOI:** 10.1186/s12889-023-17031-6

**Published:** 2023-10-25

**Authors:** Vita Špečkauskienė, Justina Trišauskė, Monika Grincaitė, Vilma Kriaučionienė, Aušra Petrauskienė

**Affiliations:** 1 Health Research Institute, Faculty of Public Health, Lithuanian University of Health Sciences, Kaunas, LT47181 Lithuania; 2 Department of Physics, Mathematics, and Biophysics, Faculty of Medicine, Lithuanian University of Health Sciences, Kaunas, LT 50162 Lithuania; 3 Department of Preventive Medicine, Faculty of Public Health, Lithuanian University of Health Sciences, Kaunas, LT47181 Lithuania

**Keywords:** Obesity, Overweight, Height, Weight, Anthropometry, Changes, First-graders

## Abstract

**Introduction:**

The monitoring of children’s growth plays a crucial role in assessing their growth patterns and indicating their weight status. Overweight and obesity, determined by cut-offs of body mass index (BMI), are most commonly associated with unhealthy nutrition, non-communicable diseases, and other health disorders. The World Health Organization (WHO) initiated the WHO European Childhood Obesity Surveillance Initiative (COSI) to measure routinely trends in children’s anthropometric changes, which allow intercountry comparisons within the European Region. Lithuania joined the COSI initiative. Our study aims to evaluate and compare underweight, normal and elevated weight (overweight and obesity) changes of Lithuanian first-graders during eleven years period.

**Methods:**

This study was composed according to the COSI study protocol, and it compares the five rounds of anthropometric measurements of Lithuanian first-graders (7-8-year-old) from 2008 to 2019. The main analysed variables were weight and height; calculated BMI, weight-for-age (W/A), height-for-age (H/A) and body mass index-for-age (BMI/A) z-scores. Changes of the indicators were evaluated according to the International Obesity Task Force (IOTF) cut-offs and WHO child growth standards, grouped by 4 estimates: underweight (thinness), normal weight, overweight and obesity. All comparisons were performed between age groups, gender and COSI round year.

**Results:**

The mean values for weight, height, W/A and H/A Z-scores were significantly higher for both age and gender groups when comparing later COSI round years (2016 or 2019) to earlier years (2008–2013, in some cases 2016 is included in this range). Time trends of the WHO growth standards and IOTF cut-offs indicate significant decrease among 7-year-old overweight group for boys and girls. Also, a significant decrease was indicated among boys aged 8 years in the overweight group according to the WHO definitions. The only significant increase in trend were expressed in girl’s group with obesity aged 8 years according to IOTF cut-offs.

**Conclusion:**

The prevalence of overweight and obesity of first-grade children in Lithuania indicates positive changes, that is the proportion of children with elevated body weight decreased, during eleven-year period. However, it is important to continue the monitoring of children’s growth tendencies.

## Introduction

The monitoring of children’s growth indicators, both nationally and internationally, is a very important area of public health. It is possible to assess children’s growth trends and identify emerging problems in time, such as underweight, overweight, and obesity, by continuously carrying out surveys and analysis of children’s anthropometric indicators. It is important to monitor weight because nutritional status can have the impact on child’s development, physical and mental health. In addition, the anthropometric indicators can reflect the quality of child nutrition and physical activity, quality of health care services, environmental conditions, implemented prevention and healthy lifestyle programs, and their need [1–3]. Low height and low body mass index (BMI) usually is caused by poor living conditions, malnutrition, and it is associated with child’s poor growth and development [4, 5], increased risk of infectious diseases [6, 7], anemia [8], psychological and behavioural problems [9]. Furthermore, overweight and obesity reflect poor nutritional conditions, an unbalanced diet rich in calories and low in nutrients [10]. Increased BMI is associated with non-communicable diseases like cardiovascular diseases [11–13], type 2 diabetes, and cancer, also it affects mental health [12], etc. Moreover, anthropometric indicators in childhood are related to indicators in older age [14, 15].

To routinely measure trends in overweight and obesity of primary schoolchildren and to form a correct understanding of the epidemic in this population group and to permit intercountry comparisons within the European Region, in 2006 the WHO initiated the WHO European Childhood Obesity Surveillance Initiative (COSI). Initially, 13 countries of the WHO European region contributed to the initiative. In 2018–2020, 45 countries participated in the survey [16]. This survey in all countries was conducted according to a standardized methodology and a unified study protocol [1]. The Lithuanian University of Health Sciences has been involved in the initiative from its planning and the first data collection round was conducted in 2008.

Over the past few decades, the epidemiological situation of childhood overweight and obesity has changed across the countries. In the developed countries overweight and obesity rates were the highest, and obesity was being identified as an epidemic [17–19], however, obesity has reached a plateau in some countries, and the rates of children with elevated BMI has stopped rising [20–23]. Based on COSI data, the prevalence of overweight and obesity among boys and girls decreased in Southern Europe countries, and it remained stable or slightly increased in Northern European and Eastern European countries. Among boys, the highest decrease in overweight (including obesity) was observed in Portugal (from 40.5% to 2007/2008 to 28.4% in 2015/2017) and in Greece for obesity (from 30.5% to 2009/2010 to 21.7% in 2015/2017) [24]. Notwithstanding these tendencies should be treated with great caution as they may be short-term [25]. World Obesity Federation (WOF) analysis shows that in 196 countries, 8 out of 10 have less than a 10% chance of keeping levels of childhood obesity no higher in 2025 than they were in 2010–2012 [26]. It is predicted that in 2030 there will be 254 million children with obesity age 5–19 years [26]. For Lithuania, the chances of meeting the WHO 2025 target (keeping levels of childhood obesity no higher than in 2020 − 2012) is only 9% [26]. To ensure the decreasing trends of overweight and obesity, it is important to introduce and implement effective policies and interventions and to continue monitoring the situation systematically.

WHO pays special attention to children’s nutrition and weight problems. Therefore, it has developed a comprehensive integrated package of recommendations to address childhood obesity [15], policy actions to increase physical activity globally [27], provided evidence-based public health recommendations for children, adolescents, adults and older adults on the amount of physical activity and sedentary behaviour [28] and took other significant initiatives in order to protect public health. These policies are usually targeting the main causes of overweight and obesity – to reduce the consumption of poor quality and high in calories food, to increase consumption of vegetables and fruits, and reduce sedentary lifestyle by increasing physical activity.

Children’s growth is assessed using anthropometric measurements (height and weight). These measurements are evaluated using standards. The most popular are WHO child growth standards (2007) [29, 30] and International anti-obesity working groups (International Obesity Task Force, IOTF) (2000, 2007) [31, 32]. For data analysis of this survey, we used both definitions. In Lithuania, the percentile method is used to assess the growth of children in the health care practice. Children’s growth is assessed using the one-dimensional and two-dimensional growth curves (according to age and gender) prepared by Tutkuviene in 1995. One-dimensional percentile diagrams can be used to assess children’s height, weight, head circumference according to age, two-dimensional percentile diagrams assess the variation of weight depending on height [33].

As mentioned above, monitoring children’s growth is an important area of public health in every country, Lithuania is no exception. Participation in the COSI initiative makes it possible to assess changes in the anthropometric indicators of Lithuanian first-graders from 2008 to 2019. In addition, the application of two international standards allows them to be compared with each other. Therefore, the **aim** of this study is to analyse the changes of anthropometric indicators of first-graders of Lithuania in 2008–2019 year by using two different international definitions.

## Methods

### Study design and subjects

This cross-sectional study was conducted following the international COSI protocol and data collection procedures approved by WHO. Detailed methodology published elsewhere [1].

According to the COSI study protocol, at least 2800 children of the target age group should be measured in each country. To achieve this number in each survey round the sample was 5800 first grade children. A semi-longitudinal design, meaning that a new cross-sectional sample of children was selected in all study rounds. Nationally representative samples of children were drawn. Three-stage cluster sampling was applied using the county as the primary sampling unit, school as the secondary and class as the tertiary sampling unit. Two age groups (7.0-7.9 and 8.0-8.9 years) were taken to be representative of the total population in these age groups. In each survey round, the sample of schoolchildren representing Lithuania was made in proportion to the number of children of selected age in each county at the beginning of the year (based on the data of Lithuania Statistics). Stratification was applied by county and level of urbanisation as differences across strata were expected. Schools from all ten counties of Lithuania were randomly selected (from the list of schools received from the Ministry of Education, Science and Sport). All first-grade classes of selected schools were included in the study and all schoolchildren in the classes were invited to participate. In this research data of younger than 7 and older than 9-year old children were not included in the data analysis. Data were collected for up to 10 weeks in each survey year. All five rounds of the survey were conducted between April and May. Description of the participation rates are provided in Table 1.

**Table 1:** Number of participants (N) and response rates (%) for each COSI round year

Year	Round	Total measured anthropometrically	Response rate (%)	Included in data analysis
				Boys N (%) – Girls N (%) – Total N
2008	1	4939	81.7	2527 (51.9) – 2341 (48.1) – 4868
2010	2	4986	80.8	2461 (50.1) – 2455 (49.9) – 4916
2013	3	3936	67.9	1951 (49.9) – 1956 (50.1) – 3907
2016	4	3920	70.8	1945 (50.6) – 1899 (49.4) – 3844
2019	5	3261	57.0	1649 (51.0) – 1582 (49.0) – 3231

### Anthropometric measurements

The anthropometric data were collected using a COSI Child’s Record Form (filled out by the examiner): name, surname, gender, date of birth, date of measurement, the reason given by a child who did not give permission to be measured, and records of measured weight and height. Children were asked to take off their shoes, heavy clothes, and items such as wallet, keys, mobile phone, hair ornaments or braids, etc. The clothes worn by a child while being measured were noted in Child Record Form [1, 34]. During data analyses, body weight was adjusted for the weight of the clothes worn by the children when they were measured. The same anthropometric equipment was used in the measurements at all schools during each study year. Measurements were carried out by the SECA’s Portable Medical Scales and Height Boards. Body weight was measured in kilograms and recorded to the nearest 100 g (0.1 kg) unit. Height was measured in centimetres and the reading taken to the last completed 1 mm (0.1 cm) [34]. BMI was calculated using the formula: weight (kg) divided by height squared (m^2^). Calculated BMI values were evaluated according to the International Obesity Task Force (IOTF) cut-offs [31, 32, 35] and WHO child growth standards [29, 30]. The WHO Anthro Software (anthropometric calculator module) was used to calculate z-scores for the assessment of individual child’s growth [36].

### Statistical analysis

Statistical analysis was performed in SPSS Statistics version 27 (IBM, Armonk, NY, USA). Difference between compared groups and relation between variables considered to be statistically significant if p < 0.05. Prevalence estimates of underweight (thinness), normal weight, overweight and obesity groups are presented as totals and percentages by age group and gender in each COSI round year. Means and standard deviations (SD) were calculated for all measurements (weight and height) and anthropometric indices (BMI, W/A, H/A and BMI/A Z-scores) by age group and gender in each COSI round year. Each COSI round year dataset, having these six continuous variables, were tested for normality by age group and gender using Kolmogorov-Smirnov test. Weight and BMI for both age groups and gender were not normally distributed in all COSI round years. These variables were transformed to attain normality and their transformed values were used for comparisons between COSI round years. The inverse transformation for weight and 1/square transformation for BMI were performed. A one-way ANOVA with Tukey HSD *Post-hoc* test was performed to assess significant differences across COSI round years by gender for both age groups. Overweight and obesity trend by COSI round year was performed using a Kendall’s Tau relation analysis test.

## Results

The mean values for weight and W/A Z-score are presented in Table 2. Statistically significant differences between weight and COSI round year were found for 7-year-old boys and girls and for 8-year-old girls. Also, statistically significant differences were found among W/A Z-score and COSI round years in all the analysed groups. One-way ANOVA analysis showed that the mean of weight and W/A Z-score for 7-year-old boys and girls were significantly higher for year 2016 or 2019 compared to earlier years (2008–2013). The same tendency can be spotted for weight of 8-year-old girls and for W/A Z-score of 8-year-old boys and girls.

**Table 2:** Means of weight and weight-for-age Z-score of boys and girls aged 7–8 years, by COSI round year

Age group and COSI round year	Mean Weight (SD), (kg)		Mean W/A Z-score (SD)	
	Boys	Girls	Boys	Girls
7-year-old	*	*	*	*
2008	27.7 (5.4)^a^	27.0 (5.4)^a^	0.68 (1.18)^a^	0.56 (1.06)^a^
2010	27.7 (5.4)^b^	27.0 (5.4)^b^	0.68 (1.19)^b^	0.54 (1.09)^b^
2013	27.8 (5.6)^c^	27.0 (5.4)^c^	0.69 (1.24)^c^	0.53 (1.09)^c^
2016	28.5 (5.7)^a,b,c^	27.3 (5.5)^a,b,c^	0.83 (1.23)^a,b,c^	0.59 (1.08)
2019	28.1 (5.6)	27.9 (5.7)	0.75 (1.22)	0.70 (1.1)^a,b,c^
8-year-old		*	*	*
2008	29.2 (5.9)	28.0 (5.2)^a,b^	0.62 (1.21)^a^	0.41 (1.03)^a,b^
2010	29.0 (5.7)	28.1 (5.6)^c,d^	0.58 (1.19)^b^	0.40 (1.07)^c,d^
2013	29.5 (6.3)	28.3 (5.7)^e^	0.70 (1.27)	0.46 (1.09)
2016	30.1 (6.3)	29.1 (6.4)^a,c^	0.80 (1.23)^a,b^	0.58 (1.14)^a,c^
2019	29.6 (6.0)	29.3 (6.3)^b,d,e^	0.72 (1.18)	0.61 (1.13)^b,d^

Body weight was adjusted for clothes worn when measured and children with a W/A Z-score < − 6 or > + 5 were excluded

*Statistically significant differences of mean values between COSI round years for the indicated gender and age group (one-way ANOVA; p-value < 0.005)

^a,b,c,d,e^ Within each gender and age group, mean values that share the same superscript letter statistically significantly differ from each other (Tukey HSD *post hoc* test; p-value < 0.05)

The mean values for height and H/A Z-score are presented in Table 3. Statistically significant differences between height, H/A Z-score and COSI round year were found for 7-8-year-old boys and girls. The tendency of significantly higher boys and girls, and higher H/A Z-score are observed for later COSI round years (2016–2019) compared to earlier COSI round years (2008–2013).

**Table 3:** Means of height and height-for-age Z-score of boys and girls aged 7–8 years, by COSI round year

Age group and COSI round year	Mean Height (SD), (cm)		Mean H/A Z-score (SD)	
	Boys	Girls	Boys	Girls
7-year-old	*	*	*	*
2008	129.2 (5.6)^a,b^	128.6 (5.5)^a^	0.68 (1.00)^a,b^	0.72 (0.95)^a^
2010	129.5 (5.7)^c,d^	128.5 (5.7)^b,c^	0.75 (1.00)^c,d^	0.72 (0.99)^b^
2013	129.4 (5.9)^e,f^	128.5 (5.9)^d^	0.73 (1.05)^e,f^	0.69 (1.03)^c^
2016	130.5 (5.8)^a,c,e^	128.9 (5.5)^e^	0.89 (1.03)^a,c,e^	0.76 (0.95)^d^
2019	130.7 (5.7)^b,d,f^	129.7 (5.6)^a,b,c,d,e^	0.92 (1.01)^b,d,f^	0.88 (0.96)^a,b,c,d^
8-year-old	*	*	*	*
2008	131.8 (5.7)^a,b^	130.9 (5.3)^a,b^	0.60 (1.00)^a,b^	0.57 (0.92)^a,b^
2010	131.7 (5.7)^c,d^	130.9 (5.5)^c,d^	0.60 (1.00)^c,d^	0.58 (0.95)^c,d^
2013	132.0 (6.0)^e,f^	130.9 (6.0)^e,f^	0.68 (1.05)^e^	0.58 (1.04)^e,f^
2016	133.0 (5.8)^a,c,e^	132.1 (5.9)^a,c,e^	0.84 (1.02)^a,c^	0.76 (1.01)^a,c,e^
2019	133.1 (6.0)^b,d,f^	132.3 (5.5)^b,d,f^	0.84 (1.06)^b,d,e^	0.78 (0.94)^b,d,f^

Children with a H/A Z-score < − 6 or > + 6 were excluded

*Statistically significant differences of mean values between COSI round years for the indicated gender and age group (one-way ANOVA; p-value < 0.001)

^a,b,c,d,e,f^ Within each gender and age group, mean values that share the same superscript letter statistically significantly differ from each other (Tukey HSD *post hoc* test; p-value < 0.05)

The mean values for BMI and BMI/A Z-score are presented in Table 4. Analysing mean BMI and BMI/A Z-score significant results were found only between the mean BMI in 7-year-old girls and COSI round years, indicating lower BMIs in earlier COSI round years (2010 and 2013) compared to later COSI round year (2019). No significant differences were found among the mean rates for BMI and BMI/A Z-score in other study groups compared to COSI round year.

**Table 4:** Means of BMI and BMI-for-age Z-score of boys and girls aged 7–8 years, by COSI round year

Age group and COSI round year	Mean BMI (SD), (kg/m^2^)		Mean BMI/A Z-score (SD)	
	Boys	Girls	Boys	Girls
7-year-old		*		
2008	16.5 (2.2)	16.3 (2.4)	0.36 (1.20)	0.19 (1.12)
2010	16.4 (2.4)	16.2 (2.4)^a^	0.31 (1.28)	0.17 (1.14)
2013	16.5 (2.4)	16.2 (2.4)^b^	0.34 (1.31)	0.16 (1.14)
2016	16.6 (2.5)	16.3 (2.4)	0.40 (1.32)	0.21 (1.14)
2019	16.4 (2.4)	16.5 (2.6)^a,b^	0.26 (1.31)	0.26 (1.19)
8-year-old				
2008	16.7 (2.5)	16.3 (2.3)	0.34 (1.28)	0.10 (1.10)
2010	16.6 (2.4)	16.3 (2.4)	0.29 (1.26)	0.08 (1.14)
2013	16.8 (2.7)	16.4 (2.5)	0.39 (1.36)	0.17 (1.13)
2016	16.9 (2.7)	16.6 (2.7)	0.44 (1.30)	0.20 (1.21)
2019	16.7 (2.5)	16.6 (2.8)	0.30 (1.28)	0.22 (1.22)

Body weight was adjusted for clothes worn when measured and children with a BMI/A Z-score < − 5 or > + 5 were excluded

*Statistically significant differences of mean values between COSI round years for the indicated gender and age group (one-way ANOVA; p-value < 0.02)

^a,b^ Within each gender and age group, mean values that share the same superscript letter statistically significantly differ from each other (Tukey HSD *post hoc* test; p-value < 0.05)

**Fig. 1:**
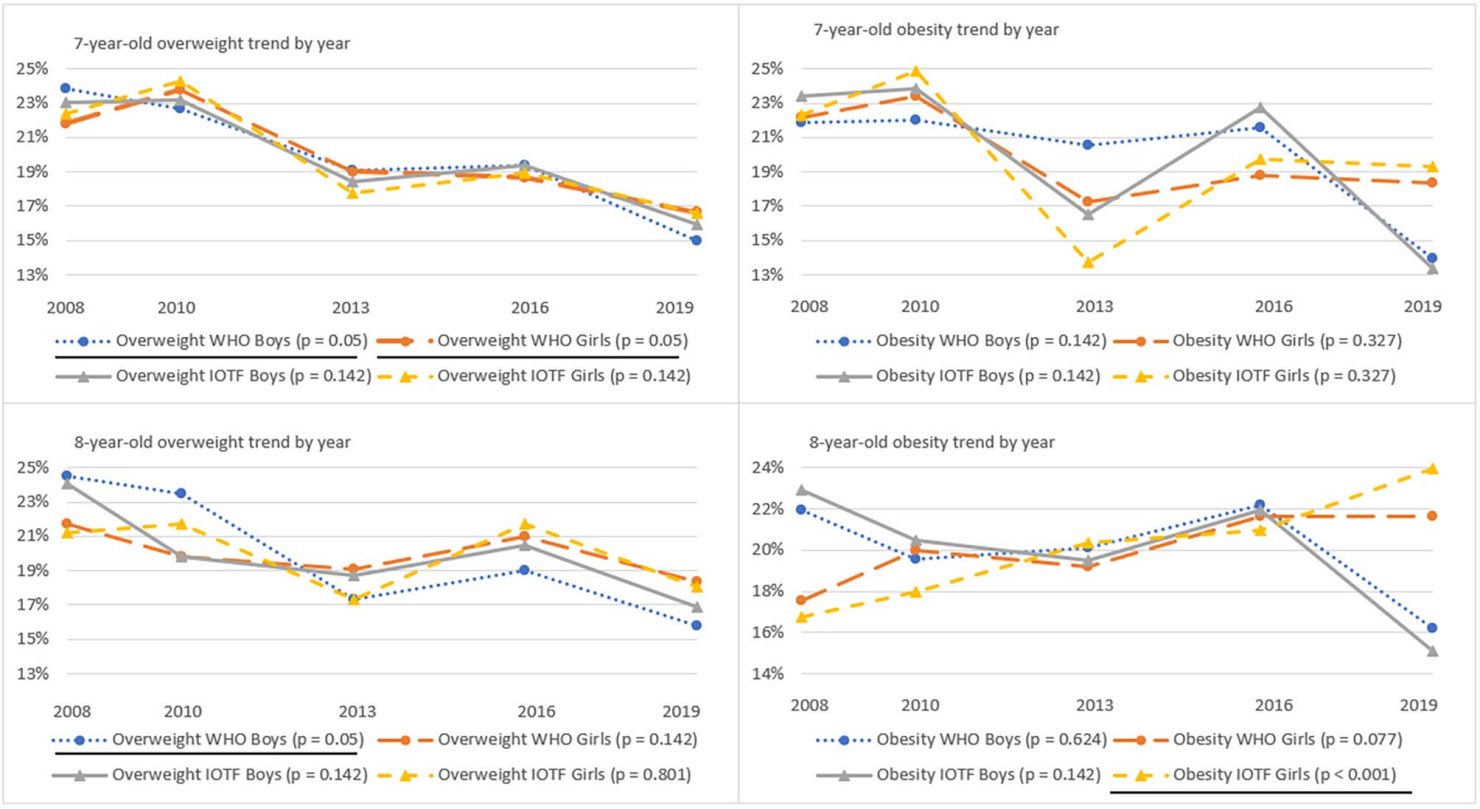
Overweight and obesity trends of 7-8-year-old children by WHO and IOTF cut-offs in five rounds of the survey

The time trends of WHO and IOTF cut-offs (Fig. 1) indicate the prevalence of overweight and obesity among children aged 7-8-years. The significant decrease is indicated among 7-year-old overweight group for boys and girls according to both cut-offs (p = 0.05). The significant decrease is also spotted among boys aged 8 years in overweight group according to WHO cut-offs (p = 0.05). The only significant increase in trend were indicated in the girl’s group with obesity aged 8 years according to IOTF cut-offs (p < 0.001), the WHO trend line in this group is close, but not significant (p = 0.077).

The following Tables 5 and 6 present the overall nutritional status divided into 4 groups of 7-8-year-old children by gender in five COSI rounds of the survey according to the WHO and IOTF cut-offs separately.

**Table 5:** Nutritional status of 7-8-year-old children by gender and COSI round year according to WHO cut-offs

Age group and study year	Underweight		Normal weight		Overweight		Obesity		Total	
	Boys	Girls	Boys	Girls	Boys	Girls	Boys	Girls	Boys	Girls
	Frequency (percentage)									
7-year-old										
2008	21 (1.3)	34 (2.1)	1224 (73.9)	1268 (76.9)	256 (15.5)	229 (13.9)	155 (9.4)	118 (7.2)	1656 (50.1)	1649 (49.9)
2010	38 (2.3)	38 (2.1)	1204 (73.3)	1380 (77.0)	244 (14.9)	250 (13.9)	156 (9.5)	125 (7.0)	1642 (47.8)	1793 (52.2)
2013	38 (2.9)	27 (2.0)	936 (70.7)	1033 (76.4)	205 (15.5)	200 (14.8)	146 (11.0)	92 (6.8)	1325 (49.5)	1352 (50.5)
2016	22 (1.7)	27 (2.1)	888 (69.9)	978 (75.2)	208 (16.4)	196 (15.1)	153 (12.0)	100 (7.7)	1271 (49.4)	1301 (50.6)
2019	25 (2.4)	19 (1.8)	761 (72.8)	772 (72.6)	161 (15.4)	175 (16.5)	99 (9.5)	98 (9.2)	1046 (49.6)	1064 (50.4)
8-year-old										
2008	22 (2.5)	21 (3.0)	622 (71.4)	538 (77.8)	143 (16.4)	90 (13.0)	84 (9.7)	43 (6.2)	871 (55.7)	692 (44.3)
2010	17 (2.1)	17 (2.6)	590 (72.0)	514 (77.6)	137 (16.7)	82 (12.4)	75 (9.2)	49 (7.4)	819 (55.3)	662 (44.7)
2013	18 (2.9)	7 (1.2)	430 (68.7)	471 (78.0)	101 (16.1)	79 (13.1)	77 (12.3)	47 (7.8)	626 (50.9)	604 (49.1)
2016	11 (1.6)	14 (2.3)	467 (69.3)	444 (74.3)	111 (16.5)	87 (14.6)	85 (12.6)	53 (8.9)	674 (53.0)	598 (47.0)
2019	12 (2.0)	16 (3.1)	437 (72.5)	373 (72.0)	92 (15.3)	76 (14.7)	62 (10.3)	53 (10.2)	603 (53.8)	518 (46.2)

Children with a BMI/A z-score <-5 or > + 5 were excluded

**Table 6:** Nutritional status of 7-8-year-old children by gender and COSI round year according to IOTF cut-offs

Age group and study year	Underweight		Normal weight		Overweight		Obesity		Total	
	Boys	Girls	Boys	Girls	Boys	Girls	Boys	Girls	Boys	Girls
	Frequency (percentage)									
7-year-old										
2008	97 (5.9)	147 (8.9)	1253 (75.7)	1172 (71.1)	201 (12.1)	218 (13.2)	105 (6.4)	112 (6.8)	1656 (50.1)	1649 (49.9)
2010	126 (7.7)	181 (10.1)	1207 (73.5)	1250 (69.7)	202 (12.3)	237 (13.2)	107 (6.5)	125 (7.0)	1642 (47.8)	1793 (52.2)
2013	117 (8.8)	154 (11.4)	973 (73.4)	956 (70.7)	161 (12.2)	173 (12.8)	74 (5.6)	69 (5.1)	1325 (49.5)	1352 (50.5)
2016	106 (8.3)	126 (9.7)	894 (70.3)	891 (68.5)	169 (13.3)	185 (14.2)	102 (8.0)	99 (7.6)	1271 (49.4)	1301 (50.6)
2019	98 (9.4)	106 (10.0)	749 (71.6)	699 (65.7)	139 (13.3)	162 (15.2)	60 (5.7)	97 (9.1)	1046 (49.6)	1064 (50.4)
8-year-old										
2008	68 (7.8)	82 (11.9)	649 (74.5)	500 (72.3)	107 (12.3)	82 (11.9)	47 (5.4)	28 (4.0)	871 (55.7)	692 (44.3)
2010	77 (9.4)	85 (12.8)	612 (74.7)	463 (69.9)	88 (10.8)	84 (12.7)	42 (5.1)	30 (4.5)	819 (55.3)	662 (44.7)
2013	60 (9.6)	60 (9.9)	443 (70.8)	443 (73.4)	83 (13.3)	67 (11.1)	40 (6.4)	34 (5.6)	626 (50.9)	604 (49.1)
2016	46 (6.8)	76 (12.7)	492 (73.0)	403 (67.4)	91 (13.5)	84 (14.0)	45 (6.7)	35 (5.9)	674 (53.0)	598 (47.0)
2019	44 (7.3)	68 (13.1)	453 (75.1)	340 (65.6)	75 (12.4)	70 (13.5)	31 (5.2)	40 (7.7)	603 (53.8)	518 (46.2)

Children with a BMI/A z-score <-5 or > + 5 were excluded

It is worth mentioning that the prevalence of children with obesity was higher when using WHO cut-offs and the prevalence of children with underweight was higher when using IOTF cut-offs for both age group children.

## Discussion

The mean weight, height, W/A Z-score and H/A Z-score of 7-8-years-old boys and girls (except mean weight of 8-year-old boys) were significantly higher in later COSI round years (2016 and/or 2019) compared to earlier years (2008–2013, in some cases 2016 is included in this range). These results indicate that children, who participated in the latest COSI round, were heavier and taller. This could be explained by acceleration indicated by economic growth and better living conditions. Growth changes were observed among 7-year-old boys and girls – proportion of children with overweight decreased significantly in later COSI round years according to WHO and IOTF cut-offs. However, according to IOTF cut-offs, the proportion of 8-years-old girls with obesity increased significantly and the number of normal weight children decreased.

According to WHO standards, overweight including obesity rates among 7-year-old boys changed only form 24.82% in 2008 to 24.85% in 2019, and among girls these mentioned indicators increased from 21.05 to 25.66%. Among 8-year-old boys the prevalence of overweight including obesity decreased from 26.06 to 25.53% and from 19.21 to 24.9% among girls. According to IOTF cut-off points, overweight including obesity rates among 7-year-old boys increased form 18.49% in 2008 to 19.03% in 2019, among girls the increase was from 20.03 to 24.35%. Among 8-year-old boys the prevalence of overweight including obesity changed from 17.69 to 17.59% and from 15.9 to 21.25% among girls.

The differences between WHO growth curves and IOTF cut-offs were not pronounced, the observed overweight and obesity trends were similar. More children were identified with overweight or obesity according to WHO standards, however differences between standards were observed in previous studies [37, 38]. National standards would help to assess more accurately the problem on a national level, while international standards would help to compare the country’s situation with other countries. Some countries have their own standards and can make such comparisons [39–41]. One of the most known are the United States (US) Centers for Disease Control and Prevention (CDC) clinical growth charts [42]. In Lithuania, children’s growth is monitored on the basis of children’s growth curves, which were compiled in 1995 [33]. However, during monitoring, we see natural changes in children’s growth, acceleration, that is why there is a need to update these standards. There are no national standards for BMI assessment based on national samples. However, scientist argue, that the use of different standards complicate the comparison of studies [43].

Historically, some of the first anthropometric indicators registered in Lithuania were in 1925–1927, 1965–1967 and 1985 and are presented in the book published in 1991 by Balčiūnienė and colleagues [44]. Comparing the indicators presented by the mentioned authors with the results of our monitoring study, we see a clear trend of increasing the average height and weight of children. In almost 90 years, in Lithuania the average height of 7-year-old boys has increased by 14.1 cm, and by 13.6 cm of girls. The average height of 8-year-old children changed slightly less, boys grew by 11.2 cm, girls by 10.4 cm. During the mentioned period, the average weight of 7-8-year-old boys increased by 6.2 kg and 5.3 kg, respectively, and by 6.5 kg and 5.4 kg for 7-8-year-old girls, respectively [76]. The average BMI of both of 7-8-year-old boys and girls increased by 0.5 kg/m^2^ − 0.3 kg/m^2^ and 0.9 kg/m^2^ respectively. However, these comparisons should be taken in consideration with caution, as the methodologies and especially the sample sizes of the studies conducted between 1925 and 1985 are very different from applied by our recent study.

Lithuanian children were among the highest based on the height for age z-score data from previous COSI rounds as Northern Europe children [45]. Acceleration in Lithuania and differences from other countries could be related to genetic factors and improved life conditions and environment [46, 47]. The Netherlands is considered a country with some of the tallest people, but researchers note that after 150 years of growth, they have stabilized, because the Dutch children may have reached the optimal height distribution [48]. However, another explanation is possible. Scientists hypothesize that poor living conditions, insufficient food and low availability of healthcare could be related to lower height. The unhealthy food and low nutrient value food is highly prevalent among populations in more developed countries, so it could be related to reduced quality of living conditions and as a result – lower achieved height [48]. Some studies showed that children with obesity were significantly taller during pre-pubertal years when compared to their normal-weight peers, but they tended to lose their pre-pubertal growth advantage over time. However, being a child with obesity did not lead to any advantage on adult height [19, 49, 50]. Therefore, the BMI should be taken into consideration. Larger gain in height than in BMI means healthy changes, for instance this was observed among girls in South Korea, Vietnam, and some central Asian countries, and among boys in central and western Europe, gaining too little height and too much weight for their height occurred in many countries in sub Saharan Africa, New Zealand, and the USA for boys and girls [51].

Despite the decreasing trends at national level between 2008 and 2019, the increase of the prevalence of overweight including obesity between 2008 and 2016 in Lithuania was the highest among COSI countries. The increasing trends were also observed among boys in Latvia, Norway, Bulgaria and Czech republic and among girls in Latvia and Bulgaria [24].

In previous COSI rounds decreasing trends were observed in other European countries - Greece, Italy, Portugal, Spain and Slovenia [16, 24, 52]. In 2018–2020, during the 5th round of COSI, 29% of children aged 7–9 years were living with overweight (including obesity) according to WHO definitions (31% among boys and 28% among girls). The highest prevalence of overweight among children was observed in Cyprus, Greece, Spain and Italy, the lowest in Tajikistan, Denmark, Israel and Kazakhstan [16]. It was observed that in COSI countries the prevalence of overweight was higher among boys. However, significant decrease in prevalence of overweight among boys and girls in Malta, among boys in San Marino, and among girls in Italy was observed [16, 52].

Decreasing trends in the prevalence of obesity between the 1st round of COSI (2007–2008) and the 4th round (2015–2017) were observed in Italy, Slovenia, Portugal, Greece and Spain for both boys and girls, and in Ireland, Norway and Bulgaria for girls only [24]. Comparison of the 5th round obesity prevalence with data from the 4th round found that the only significant changes were an increase in the prevalence among boys in Georgia and a decrease among boys in San Marino and girls in Malta. An increase among boys and girls was observed in Sweden [16, 52]. In 2018–2020, the prevalence of obesity among children aged 7–9 years in the 33 COSI countries was 12% (14% among boys and 10% among girls). Obesity rates were the highest in Cyprus, Italy, Greece and Spain, and the lowest in Tajikistan, Denmark, Kazakhstan and Israel [16].

Decreasing overweight and obesity trends, especially in countries with the highest rates, could be related to changes in health-related policies in countries. For instance, a good example could be Portugal where the downward trend in the prevalence of childhood overweight could be related to high-level political engagement at national, regional and local levels [3]. In Portugal in 2007, the Ministry of Health initiated a Platform against Obesity [3], in 2012 – a National Programme for Promotion of Healthy Eating [3, 53] and in 2017 – an Integrated Strategy for the Promotion of Healthy Eating [3, 54]. In Italy, National Prevention Plan 2020–2025 included specific programs for health promotion in schools, active communities, and protection of the well-being of women and children in the 1000-day period from conception to a child’s second birthday [3, 55]. In Malta, National Obesity Strategy was initiated in 2012 as a result of high prevalence of overweight and obesity among children [3, 56]. In Latvia, Georgia, Turkey and Bulgaria healthier school environments were created by improving nutritional standards for school food and restricting availability of sweet, salty and high in calories food and beverages [3].

In Lithuania positive changes in organization of nutrition in children’s educational institutions (preschools, schools, camps, children’s social care homes) were also implemented. Meals of children are organized in accordance with the description of the procedure, approved by the Minister of Health of the Republic of Lithuania in 2011 November 11 by order no. V-964 “On approval of the description of the procedure for organizing children’s meals”. The aim of this document is to ensure healthy children’s nutrition, food safety and the best quality, in order to meet the physiological needs of children’s nutrients and to develop healthy eating skills. Document describes products that are recommended and prohibited to use during food production process. It is recommended to use ecological, local food products, etc. Preference is given to food preparation methods that conserve nutritional properties (such as stewing). Vegetables and fruits (recommended seasonal, fresh) must be served during main and additional meals every day. The hot main meal should be high in proteins (meat, poultry, fish, eggs, legumes, milk, and dairy products). Salt used for cooking should contain iodine. Hygienic conditions for free drinking water must be created [57]. Children’s educational institutions are encouraged to participate in the program of promoting the consumption of fruits, vegetables, milk, and dairy products. The program is funded by European Union (EU) and state budget of the Republic of Lithuania [58]. Regardless of the family’s income, free school lunches are provided to all preschool, first and second grade children. Older children have the right to free meals if the average monthly income per one person in family is less than 1.5 amounts of the state-supported income [59]. In addition, the Ministry of Health of the Republic of Lithuania has prepared Healthy and Sustainable Nutrition Recommendations for different age groups [60].

Decreasing trends, especially in countries with the highest rates of overweight and obesity, could be related to habit changes of consumption of products high in calories and sugar, increased consumption of vegetables and fruits, greater physical activity, less sedentary behaviour, healthier environment [61, 62], and parenting practices [61–63]. By controlling and changing these factors, the anthropometric indicators of the country’s children can be affected. Reasons of lifestyle and cultural changes of nutrition in countries can be difficult to determine. Some of them may occur due to changes in health policy or programs that affect the living environment – for example, school-based interventions for preventing childhood obesity [64] and school food environment policies [65]. Other interventions and policies like sugar taxes [66], strict regulation on food marketing for children [67], calorie labelling on menus and food assistance programs [68, 69] could be effective measures to control rising rates of children overweight and obesity. It is clear that elevated body weight of children is a complex issue that requires multifactorial and sustainable solutions.

It is worth to mention the weaknesses of this study. Firstly, a tendency of decrease in participation rates in epidemiological studies was observed, with no exception to our study, where the response rates also decreased (Table 1). According to subjective examiners observation, it was noted that some children with overweight and obesity had no parental consent for the child to be measured and refused to participate in the study. This pose a risk that the prevalence of overweight and obesity in Lithuanian children is a bit underestimated. Also, during the period of the survey the requirements of Lithuanian Bioethics committee has changed and in recent years the written informed consent of two parents had to be obtained. Secondly, the sample size of 7- and 8-year-old children were not equal. Thirdly, during this study only 7-8-years-old children were measured, other age groups were not represented.

However, Lithuania’s consistent participation in the COSI initiative and conducting research in accordance with a strict international protocol allows reliable, regular, and valid data collection, systematic and objective monitoring of changes in children’s growth. These COSI results are important not only from an epidemiological point of view but can also be used in the implementation of the country’s public health policy, indicating time when appropriate actions should be taken in order to control the overweight and obesity issue in children population.

## Conclusion

Although the proportion of the first-grade children with elevated body weight decreased in Lithuania, it is important to continue monitoring growth tendencies, present the results, adopt appropriate policies, and improve the children’s environment by ensuring healthy growth of children.

## Data Availability

The datasets used and/or analysed during the current study are available from the corresponding author on reasonable request.

## List of abbreviations

BMI: Body mass index
WHO: World Health Organization
COSI: European Childhood Obesity Surveillance Initiative
WOF: World Obesity Federation
IOTF: International Obesity Task Force
W/A: weight-for-age
H/A: height-for-age
BMI/A: body mass index-for-age
EU: European Union
SD: standard deviation

## References

[1] Breda J, McColl K, Buoncristiano M, Williams J, Abdrakhmanova S, Abdurrahmonova Z, et al. Methodology and implementation of the WHO European Childhood Obesity Surveillance Initiative (COSI). Obes Rev. 2021;22(S6):e13215. 34738283 10.1111/obr.13215

[2] Physical Status. : the Use and Interpretation of Anthropometry. Report of a WHO Expert Committee. WHO Technical Report Series, No. 854. Geneva: World Health Organization; 1995. 8594834

[3] Breda J, Farrugia Sant’Angelo V, Duleva V, Galeone D, Heinen MM, Kelleher CC, et al. Mobilizing governments and society to combat obesity: reflections on how data from the WHO European Childhood Obesity Surveillance Initiative are helping to drive policy progress. Obes Rev. 2021;22(S6):e13217. 34378847 10.1111/obr.13217

[4] Gahagan S. Failure to thrive: a consequence of undernutrition. Pediatr Rev. 2006;27(1):e1–11. 16403734 10.1542/pir.27-1-e1

[5] Sares-Jäske L, Grönqvist A, Mäki P, Tolonen H, Laatikainen T. Family socioeconomic status and childhood adiposity in Europe - A scoping review. Prev Med. 2022;160:107095. 35594926 10.1016/j.ypmed.2022.107095

[6] Scrimshaw NS, SanGiovanni JP. Synergism of nutrition, Infection, and immunity: an overview. Am J Clin Nutr. 1997;66(2):464S–77. 9250134 10.1093/ajcn/66.2.464S

[7] Albers R, Bourdet-Sicard R, Braun D, Calder PC, Herz U, Lambert C, et al. Monitoring immune modulation by nutrition in the general population: identifying and substantiating effects on human health. Br J Nutr. 2013;110(S2):1–30.23902657 10.1017/S0007114513001505

[8] Wu J, Hu Y, Li M, Chen J, Mao D, Li W et al. Prevalence of anemia in Chinese children and adolescents and its associated factors. Int J Environ Res Public Health. 2019;16(8). 10.3390/ijerph16081416 PMC6518082 31010238

[9] Cimino S, Cerniglia L, Almenara CA, Jezek S, Erriu M, Tambelli R. Developmental trajectories of body mass index and emotional-behavioral functioning of underweight children: a longitudinal study. Sci Rep. 2016;6:20211. 26806123 10.1038/srep20211 PMC4726243

[10] Popkin BM, Corvalan C, Grummer-Strawn LM. Dynamics of the double burden of malnutrition and the changing nutrition reality. Lancet. 2020;395(10217):65–74. 31852602 10.1016/S0140-6736(19)32497-3 PMC7179702

[11] Friedemann C, Heneghan C, Mahtani K, Thompson M, Perera R, Ward AM. Cardiovascular Disease risk in healthy children and its association with body mass index: systematic review and meta-analysis. BMJ. 2012;345:e4759. 23015032 10.1136/bmj.e4759 PMC3458230

[12] World Health Organization. Regional Office for Europe. WHO European Regional Obesity Report 2022 [Internet]. World Health Organization. Regional Office for Europe. ; 2022 [cited 2023 Feb 28]. Available from: https://apps.who.int/iris/handle/10665/353747.

[13] McPhee PG, Singh S, Morrison KM. Childhood obesity and Cardiovascular Disease Risk: working toward solutions. Can J Cardiol. 2020;36(9):1352–61. 32622878 10.1016/j.cjca.2020.06.020

[14] Petkeviciene J, Klumbiene J, Kriaucioniene V, Raskiliene A, Sakyte E, Ceponiene I. Anthropometric measurements in childhood and prediction of cardiovascular risk factors in adulthood: Kaunas cardiovascular risk cohort study. BMC Public Health. 2015;15:218. 25880559 10.1186/s12889-015-1528-5 PMC4359556

[15] World Health Organization. Report of the commission on ending childhood obesity [Internet]. World Health Organization; 2016 [cited 2023 Feb 24]. Available from: https://apps.who.int/iris/handle/10665/204176.

[16] WHO, Regional Office for Europe. Report on the fifth round of data collection, 2018–2020: WHO European Childhood Obesity Surveillance Initiative (COSI) [Internet]. Copenhagen; 2022 [cited 2023 Feb 9] p. 70. Available from: https://www.who.int/europe/publications/i/item/WHO-EURO-2022-6594-46360-67071.

[17] Abarca-Gómez L, Abdeen ZA, Hamid ZA, Abu-Rmeileh NM, Acosta-Cazares B, Acuin C, et al. Worldwide trends in body-mass index, underweight, overweight, and obesity from 1975 to 2016: a pooled analysis of 2416 population-based measurement studies in 128·9 million children, adolescents, and adults. Lancet. 2017;390(10113):2627–42. 29029897 10.1016/S0140-6736(17)32129-3 PMC5735219

[18] Lobstein T, Jackson-Leach R, Moodie ML, Hall KD, Gortmaker SL, Swinburn BA, et al. Child and adolescent obesity: part of a bigger picture. The Lancet. 2015;385(9986):2510–20. 10.1016/S0140-6736(14)61746-3 PMC4594797 25703114

[19] Giglione E, Lapolla R, Cianfarani S, Faienza MF, Fintini D, Weber G, et al. Linear growth and puberty in childhood obesity: what is new? Minerva Pediatr. 2021;73(6):563–71. 10.23736/S2724-5276.21.06543-5 34309346

[20] Olds T, Maher C, Zumin S, Péneau S, Lioret S, Castetbon K, et al. Evidence that the prevalence of childhood overweight is plateauing: data from nine countries. Int J Pediatr Obes. 2011;6(5–6):342–60. 21838570 10.3109/17477166.2011.605895

[21] Miqueleiz E, Lostao L, Regidor E. Stabilisation of the trend in prevalence of childhood overweight and obesity in Spain: 2001–11. Eur J Public Health. 2016;26(6):960–3. 27335329 10.1093/eurpub/ckw087

[22] de Ruiter I, Olmedo-Requena R, Sánchez-Cruz JJ, Jiménez-Moleón JJ. Trends in child obesity and underweight in Spain by Birth Year and Age, 1983 to 2011. Rev Esp Cardiol Engl Ed. 2017;70(8):646–55. 28153550 10.1016/j.rec.2016.12.013

[23] NCD Risk Factor Collaboration (NCD-RisC). Worldwide trends in body-mass index, underweight, overweight, and obesity from 1975 to 2016: a pooled analysis of 2416 population-based measurement studies in 1289 million children, adolescents, and adults. Lancet Lond Engl. 2017;390(10113):2627–42. 10.1016/S0140-6736(17)32129-3 PMC5735219 29029897

[24] Buoncristiano M, Spinelli A, Williams J, Nardone P, Rito AI, García-Solano M, et al. Childhood overweight and obesity in Europe: changes from 2007 to 2017. Obes Rev off J Int Assoc Study Obes. 2021;22:e13226. 10.1111/obr.13226 34378305

[25] Ludwig DS. Epidemic childhood obesity: not yet the end of the beginning. Pediatrics. 2018;141(3):e20174078. 29483198 10.1542/peds.2017-4078 PMC5847089

[26] World Obesity. Atlas of childhood obesity October 2019. World Obesity Federation. p. 212.

[27] World Health Organization. Global action plan on physical activity 2018–2030: more active people for a healthier world [Internet]. World Health Organization; 2018 [cited 2023 Feb 24]. 101 p. Available from: https://apps.who.int/iris/handle/10665/272722.

[28] WHO guidelines on physical activity and sedentary behaviour [Internet]. [cited 2023 Feb 24]. Available from: https://www.who.int/publications-detail-redirect/9789240015128.

[29] WHO Multicentre Growth Reference Study Group. WHO Child Growth standards: Length/height-for-age, weight-for-age, weight-for-length, weight-for-height and body mass index-for-age: methods and development. Geneva: World Health Organization; 2006.

[30] Group WMGRS, de Onis M. WHO Child Growth standards based on length/height, weight and age. Acta Paediatr. 2006;95(S450):76–85. 10.1111/j.1651-2227.2006.tb02378.x 16817681

[31] Cole TJ. Establishing a standard definition for child overweight and obesity worldwide: international survey. BMJ. 2000;320(7244):1240–0. 10797032 10.1136/bmj.320.7244.1240 PMC27365

[32] Cole TJ, Flegal KM, Nicholls D, Jackson AA. Body mass index cut offs to define thinness in children and adolescents: international survey. BMJ. 2007;335(7612):194. 17591624 10.1136/bmj.39238.399444.55 PMC1934447

[33] Tutkuvienė J. Vaikų augimo ir brendimo įvertinimas. Vilnius: Meralas; 1995.

[34] World Health Organization. Regional Office for Europe. Childhood Obesity Surveillance Initiative (COSI). Data Collection procedures - mandatory and optional items (2021–2023). Copenhagen: World Health Organization; 2021. p. 51.

[35] Obesity Classification [Internet]. World Obesity Federation. [cited 2023 Feb 28]. Available from: https://www.worldobesity.org/about/about-obesity/obesity-classification.

[36] WHO Anthro Survey Analyser and other tools [Internet]. [cited 2023 Apr 4]. Available from: https://www.who.int/tools/child-growth-standards/software.

[37] Lauria L, Spinelli A, Buoncristiano M, Nardone P. Decline of childhood overweight and obesity in Italy from 2008 to 2016: results from 5 rounds of the population-based surveillance system. BMC Public Health. 2019;19(1):618. 31113403 10.1186/s12889-019-6946-3 PMC6528349

[38] Rito A, Wijnhoven TMA, Rutter H, Carvalho MA, Paixão E, Ramos C, et al. Prevalence of obesity among Portuguese children (6–8 years old) using three definition criteria: COSI Portugal, 2008. Pediatr Obes. 2012;7(6):413–22. 22899658 10.1111/j.2047-6310.2012.00068.x

[39] Al-Hazzaa HM, Alrasheedi AA, Alsulaimani RA, Jabri L, Alhowikan AM, Alhussain MH, et al. Prevalence of overweight and obesity among Saudi children: a comparison of two widely used international standards and the national growth references. Front Endocrinol. 2022;13:954755. 10.3389/fendo.2022.954755 PMC9393362 36004353

[40] Kêkê LM, Samouda H, Jacobs J, di Pompeo C, Lemdani M, Hubert H, et al. Body mass index and childhood obesity classification systems: a comparison of the French, International Obesity Task Force (IOTF) and World Health Organization (WHO) references. Rev Epidemiol Sante Publique. 2015;63(3):173–82. 26002984 10.1016/j.respe.2014.11.003

[41] Sarkkola C, Viljakainen J, de Oliveira Figueiredo RA, Saari A, Lommi S, Engberg E, et al. Prevalence of thinness, overweight, obesity, and Central Obesity in Finnish school-aged children: a comparison of National and International Reference values. Obes Facts. 2022;15(2):240–7. 34937040 10.1159/000521170 PMC9021618

[42] Growth Charts - Clinical Growth Charts [Internet]. 2022. Available from: https://www.cdc.gov/growthcharts/clinical_charts.htm.

[43] Cole TJ, Lobstein T. Exploring an algorithm to harmonize International Obesity Task Force and World Health Organization child overweight and obesity prevalence rates. Pediatr Obes. 2022;17(7):e12905. 35193166 10.1111/ijpo.12905 PMC9285550

[44] Balčiūnienė I, Naynys JV, Pavilionis S, Tutkuvienė J. Lietuvių antropologijos metmenys. Vilnius: Mokslas; 1991.

[45] Spinelli A, Buoncristiano M, Nardone P, Starc G, Hejgaard T, Júlíusson PB, et al. Thinness, overweight, and obesity in 6- to 9-year-old children from 36 countries: the World Health Organization European Childhood Obesity Surveillance Initiative—COSI 2015–2017. Obes Rev. 2021;22(S6):e13214. 34235850 10.1111/obr.13214

[46] Robinson MR, Hemani G, Medina-Gomez C, Mezzavilla M, Esko T, Shakhbazov K, et al. Population genetic differentiation of height and body mass index across Europe. Nat Genet. 2015;47(11):1357–62. 26366552 10.1038/ng.3401 PMC4984852

[47] Jelenkovic A, Sund R, Hur YM, Yokoyama Y, Hjelmborg JvB, Möller S, et al. Genetic and environmental influences on height from infancy to early adulthood: an individual-based pooled analysis of 45 twin cohorts. Sci Rep. 2016;6(1):28496. 27333805 10.1038/srep28496 PMC4917845

[48] Schönbeck Y, Talma H, van Dommelen P, Bakker B, Buitendijk SE, HiraSing RA, et al. The world’s tallest nation has stopped growing taller: the height of Dutch children from 1955 to 2009. Pediatr Res. 2013;73(3):371–7. 23222908 10.1038/pr.2012.189

[49] De Leonibus C, Marcovecchio ML, Chiavaroli V, de Giorgis T, Chiarelli F, Mohn A. Timing of puberty and physical growth in obese children: a longitudinal study in boys and girls. Pediatr Obes. 2014;9(4):292–9. 23713062 10.1111/j.2047-6310.2013.00176.x

[50] He Q, Karlberg J. Bmi in childhood and its association with height gain, timing of puberty, and final height. Pediatr Res. 2001;49(2):244–51. 11158521 10.1203/00006450-200102000-00019

[51] NCD Risk Factor Collaboration (NCD-RisC). Height and body-mass index trajectories of school-aged children and adolescents from 1985 to 2019 in 200 countries and territories: a pooled analysis of 2181 population-based studies with 65 million participants. Lancet Lond Engl. 2020;396(10261):1511–24. 10.1016/S0140-6736(20)31859-6 PMC7658740 33160572

[52] World Health Organization. Regional Office for Europe. WHO European Childhood Obesity Surveillance Initiative (COSI) Report on the fourth round of data collection, 2015–2017 [Internet]. World Health Organization. Regional Office for Europe. ; 2021 [cited 2023 Feb 9]. Report No.: WHO/EURO:2021-2495-42251-58349. Available from: https://apps.who.int/iris/handle/10665/341189.

[53] Graça P, Gregório MJ, Mendes de Sousa S, Carriço J, Correia A, Salvador C, et al. The Portuguese National Programme for the Promotion of healthy eating: 2012–2015. Public Health Panor. 2016;02(02):184–209.

[54] Graça P, Gregório MJ, de Sousa SM, Brás S, Penedo T, Carvalho T, et al. A new interministerial strategy for the promotion of healthy eating in Portugal: implementation and initial results. Health Res Policy Syst. 2018;16(1):102. 30376876 10.1186/s12961-018-0380-3 PMC6208124

[55] Ministero della Salute, Direzione Generale della Prevenzione Sanitaria. Piano Nazionale Della Prevenzione 2020–2025 [National Prevention Plan 2020–2025]. [Internet]. 2020. Available from: http://www.salute.gov.it/imgs/C_17_notizie_5029_0_file.pdf.

[56] Superintendence of Public Health. Ministry for Health, the Elderlyand Community Care.A Healthy Weight for Life. A National Strategyfor Malta 2012–2020. Msida, Malta: Superintendence of PublicHealth; 2012.

[57] V-964 Dėl Vaikų. maitinimo organizavimo tvarkos aprašo patvirtinimo [Internet]. [cited 2023 Feb 22]. Available from: https://e-seimas.lrs.lt/portal/legalAct/lt/TAD/TAIS.411986/asr.

[58] 3D-599. Dėl Vaisių ir daržovių bei pieno ir pieno produktų vartojimo skatinimo vaikų ugdymo įstaigose pro… Internet]. [cited 2023 Feb 22]. Available from: https://e-seimas.lrs.lt/portal/legalAct/lt/TAD/61200a82a22c11e7a65c90dfe4655c64/asr.

[59] X-686 Lietuvos Respublikos. socialinės paramos mokiniams įstatymas [Internet]. [cited 2023 Feb 24]. Available from: https://e-seimas.lrs.lt/portal/legalAct/lt/TAD/TAIS.279123/asr.

[60] Sveikos mitybos rekomendacijos [Internet]. [cited 2023 Feb 24]. Available from: https://sam.lrv.lt/lt/veiklos-sritys/visuomenes-sveikatos-prieziura/mityba-ir-fizinis-aktyvumas-2/sveikos-mitybos-rekomendacijos.

[61] Lee EY, Yoon KH. Epidemic obesity in children and adolescents: risk factors and prevention. Front Med. 2018;12(6):658–66. 30280308 10.1007/s11684-018-0640-1

[62] Nobles J, Summerbell C, Brown T, Jago R, Moore T. A secondary analysis of the childhood obesity prevention Cochrane Review through a wider determinants of health lens: implications for research funders, researchers, policymakers and practitioners. Int J Behav Nutr Phys Act. 2021;18(1):22. 33563281 10.1186/s12966-021-01082-2 PMC7874658

[63] Vaitkevičiūtė J, Petrauskienė A. The associations between Body Mass Index of seven- and eight-year-old children, Dietary Behaviour and Nutrition-Related Parenting practices. Med Kaunas Lith. 2019;55(1):24. 10.3390/medicina55010024 PMC6359571 30669687

[64] Wang Y, Cai L, Wu Y, Wilson RF, Weston C, Fawole O, et al. What childhood obesity prevention programmes work? A systematic review and meta-analysis. Obes Rev off J Int Assoc Study Obes. 2015;16(7):547–65. 10.1111/obr.12277 PMC4561621 25893796

[65] Micha R, Karageorgou D, Bakogianni I, Trichia E, Whitsel LP, Story M, et al. Effectiveness of school food environment policies on children’s dietary behaviors: a systematic review and meta-analysis. PLoS ONE. 2018;13(3):e0194555. 29596440 10.1371/journal.pone.0194555 PMC5875768

[66] Cabrera Escobar MA, Veerman JL, Tollman SM, Bertram MY, Hofman KJ. Evidence that a tax on sugar sweetened beverages reduces the obesity rate: a meta-analysis. BMC Public Health. 2013;13:1072. 24225016 10.1186/1471-2458-13-1072 PMC3840583

[67] World Health Organization. Regional Office for Europe. Evaluating implementation of the WHO Set of Recommendations on the marketing of foods and non-alcoholic beverages to children: progress, challenges and guidance for next steps in the WHO European Region [Internet]. World Health Organization. Regional Office for Europe; 2018 [cited 2023 Feb 14]. Report No.: WHO/EURO:2018-3299-43058-60256. Available from: https://apps.who.int/iris/handle/10665/345153.

[68] Fox AM, Horowitz CR. Best practices in Policy approaches to obesity Prevention. J Health Care Poor Underserved. 2013;24(2 0):168–92. 23727973 10.1353/hpu.2013.0097 PMC4282160

[69] Pineda E, Bascunan J, Sassi F. Improving the school food environment for the prevention of childhood obesity: what works and what doesn’t. Obes Rev. 2021;22(2):e13176. 33462933 10.1111/obr.13176

