# Correction: Changes of anthropometric indicators of Lithuanian first-graders in 2008–2019 according to International Obesity Task Force (IOTF) and World Health Organization (WHO) definitions

**DOI:** 10.1186/s12889-024-19349-1

**Published:** 2024-07-18

**Authors:** Vita Špečkauskienė, Justina Trišauskė, Monika Grincaitė, Vilma Kriaučionienė, Aušra Petrauskienė

**Affiliations:** 1 Health Research Institute, Faculty of Public Health, Lithuanian University of Health Sciences, Kaunas, LT47181 Lithuania; 2 Department of Physics, Mathematics, and Biophysics, Faculty of Medicine, Lithuanian University of Health Sciences, Kaunas, 50162 LT Lithuania; 3 Department of Preventive Medicine, Faculty of Public Health, Lithuanian University of Health Sciences, Kaunas, LT47181 Lithuania

**Correction: BMC Public Health (2023) 23:2097**

10.1186/s12889-023-17031-6

In the original publication of this article the author names were inverted. The incorrect and correct information is listed in this correction article. The original article has been updated.

Incorrect

Špečkauskienė Vita, Trišauskė Justina, Grincaitė Monika, Kriaučionienė Vilma & Petrauskienė Aušra

Correct

Vita Špečkauskienė, Justina Trišauskė, Monika Grincaitė, Vilma Kriaučionienė & Aušra Petrauskienė

